# Targeted Treatment in Childhood Epilepsy Syndromes

**DOI:** 10.1007/s11940-016-0407-4

**Published:** 2016-05-07

**Authors:** Katharina Vezyroglou, J. Helen Cross

**Affiliations:** Clinical Neurosciences, 30 Guilford St, London, WC1N 1EH UK; Great Ormond Street Hospital for Children NHS Trust, Great Ormond Street, London, WC1N 3JH UK

**Keywords:** Childhood epilepsies, Epileptic encephalopathy, Antiepileptic drugs (AED), Ketogenic diet (KD), Cannabidiol (CBD), Next generation sequencing (NGS), Targeted therapy

## Abstract

The mainstay of treatment of epilepsy has been antiepileptic drugs; however, despite the emergence of new agents, a consistent proportion remain drug-resistant. Newer AEDs show promise. However, as it becomes clear that the epilepsies are a group of diseases rather than a single disorder the prospect of targeted treatment in some may become a reality.

## Introduction

Epilepsy is a condition characterized by recurrent epileptic seizures; it is not a single condition, more accurately, we should refer to the epilepsies as there are many different underlying causes and consequently differing underlying pathophysiologies. Besides their common name, the epilepsies also share common treatment options, as traditionally, epilepsy treatment consists of therapies empirically known to raise seizure threshold rather than targeted treatments of the underlying cause. Many of the treatments widely in use today have been discovered serendipitously to have anticonvulsive properties, for example, agents such as phenobarbital or sodium valproate [1]., or fasting as a precursor of the ketogenic diet, and have since been used to treat a wide range of epilepsy syndromes. Often, their efficacy has also been validated in heterogeneous groups of patients, thus probably missing efficacy in specific epilepsy syndromes.

In recent years, great progress has been made in the understanding of mechanisms by which different anticonvulsive treatments work either by decreasing neuronal excitation or increasing neuronal inhibition at a synaptic level. This has led to the discussion of rational polytherapy, that is, utilization of a combination of drugs with different rather than similar mechanisms of action. At the same time, through the technology of next-generation sequencing, numerous gene mutations have been discovered as causal in different epilepsy syndromes, and subsequent functional studies have given insight into disease pathophysiology. Combining this knowledge, it has already been possible to identify some existing therapies as being particularly beneficiary or on the other hand contraindicated for specific epilepsy syndromes, such as for example the ketogenic diet as a targeted treatment providing an alternative fuel to glucose for children with GLUT-1 deficiency while carbamazepine or lamotrigine as sodium ion channel blockers may worsen seizures in children with SCN1A mutations (e.g., Dravet syndrome). A better understanding of pathophysiology nurtures the hope of the development of new, targeted antiepileptic treatments, and efforts are already being made in this direction.

With antiepileptic treatments available today, 30 % of epilepsy patients remain drug-resistant. This especially concerns patients with early onset epileptic encephalopathies, not only having a poor prognosis for seizure control but also an extremely poor prognosis for neurodevelopmental outcome. It is evident that this group of patients would have a lot to gain by early, targeted treatment rather than the traditional trial and error approach to epilepsy therapy. In this review, we aim to provide the existing evidence for the use of existing and some novel antiepileptic treatments as targeted treatment for specific epilepsy syndromes.

## Treatment

### Ketogenic diet

- The ketogenic diet has been used for the treatment of the epilepsies for almost 100 years [2]. It was first utilized in 1921 by Wilder at the Mayo clinic based on the discovery that fasting had been determined to help seizures. The positive effect of fasting on seizures is documented as early as in the Hippocratic collection. The Parisian physicians Guelpa and Marie first reported starvation as an epilepsy treatment in modern age in 1911. This metabolic state leads to the production of acetone and beta-hydroxybutyric acid; ketonemia, however, could also be induced by a diet containing a high proportion of fat with low carbohydrate (including protein). Even though implementation of the ketogenic diet led to encouraging results leading to it being widely used in the 1920s and 1930s, it was then overshadowed by the discovery of new antiepileptic drugs such as phenytoin and sodium valproate.
- Interest in the diet reemerged in the 1990s, and a wide range of clinical data has been published on its efficacy in treating children with drug-resistant epilepsy. Still systematic reviews criticized the lack of randomized controlled data [3–5]. The first randomized controlled trial proving the efficacy of the ketogenic diet for the management of epilepsy in children was only published in 2008 [6]. Neal et al. studied seizure control in 145 children with refractory epilepsy randomly assigning 73 children to the ketogenic diet, while the other 72 had no change to treatment. After 3 months, the mean percentage of baseline seizures was significantly lower in the diet group (*p* < 0.0001). Another randomized controlled trial conducted in 2013 [7••] proved the efficacy of the modified Atkins diet for the treatment of refractory epilepsy in children, also showing the diet to be well tolerated. The above studies showed responder rates (>50 % seizure reduction) of 38 and 52 %, respectively. In specific epilepsies, a better response may be seen. In one prospective open label study of infantile spasms, a responder rate of 76 % was seen [8]. Of patients with epilepsy due to focal malformations, 61.7 % were reported to respond to the ketogenic diet [9] and 60 % of patients with refractive epilepsy due to hypoxic ischemic encephalopathy [10].
- The ketogenic diet remains the treatment of choice for glucose transporter defects such as GLUT-1 deficiency syndrome [11•]. In this condition, delivery of glucose to the brain across the blood brain barrier is insufficient as the primary glucose transporter GLUT-1 is impaired. In its most serious form, the syndrome results in a phenotype with early onset refractory epilepsy, developmental delay, complex movement disorder and acquired microcephaly. The syndrome is due to mutations in the SLC2A1 gene on chromosome 1. With the ketogenic diet, nutritional fat is transformed into ketone bodies, which can be used as a metabolic substrate for the brain instead of glucose, it constitutes a targeted treatment for GLUT-1 deficiency. The wide phenotypic spectrum of the disorder is now becoming apparent; some children may have epilepsy responsive to antiepileptic medication. It has been shown that SLC2A1 mutations can also cause a phenotype of myoclonic astatic epilepsy (MAE) accounting for 5 % of patients with MAE [12]. SLC2A1 mutations have also been found in 1 % of a patient cohort with genetic generalized epilepsy [13]. However; Ramm-Petersen et al. demonstrated that early utilization of the diet may lead to improved neurodevelopmental outcome. It is therefore crucial that the ketogenic diet is used as first line treatment in these patients and is implemented as early as possible. These findings stress the importance of determining the genetic etiology in epilepsy patients as this might allow us to offer them early targeted treatment resulting in better outcome.
- Research is ongoing in order to understand the exact mechanism the ketogenic diet works in epilepsies other than GLUT-1 deficiency. This is important as on the one hand it might allow us to predict the patients likely to respond to the diet and on the other hand might make it possible to identify a particular therapeutic agent that could be directly administered thus releasing patients and their families of the diet’s restraints. Recently promising results have been shown for the medium chain fatty acid decanoic acid (C10) [14, 15].

#### Classical ketogenic diet

**Standard procedure**: The classical ketogenic diet is based on a ratio of fat to carbohydrate and protein, usually 3:1 or 4:1. The fat is provided by long-chain triglycerides, protein is kept to minimum requirements for growth, and carbohydrate is very restricted. Menus are calculated and foods have to be weighed to ensure accuracy. Usually the diet is started on a 2:1 ratio and gradually increased to the 4:1 ratio as tolerated over 1–2 weeks. The diet is fully supplemented with vitamins and minerals. Ketosis is monitored with urine reagent sticks or blood ketone monitors.
**Contraindications**: hyperlipidemia, renal stones, organic acid deficiency syndromes
**Complications**: hunger, vomiting, diarrhea, abdominal pain, constipation (all of which can usually be alleviated by manipulation of the diet), lack of energy, taste problems, renal stones.

Special points: the ketogenic diet is proven effective in children with epilepsy and is a highly effective targeted treatment for patients with known SLC2A1 mutations.

#### Medium-chain triglyceride (MCT) ketogenic diet

**Standard procedure**: In the MCT diet, long-chain fat is replaced by medium-chain fat from 30 % (modified MCT diet) to 60 % (traditional MCT diet) of energy intake. As MCT yields more ketones per kilocalorie of energy than long-chain fatty acids, the MCT diet allows for more protein and carbohydrates to be included in the diet. MCT is usually started at 40–45 % of energy and increased up to 60 % if necessary and tolerated. The diet is fully supplemented with vitamins and minerals.
**Contraindications**: hyperlipidemia, renal stones, organic acid deficiency syndromes
**Complications**: hunger, vomiting, diarrhea, abdominal pain, constipation (all of which can usually be alleviated by manipulation of the diet), lack of energy, taste problems, renal stones, hypoglycemia.

Special points: the MCT diet allows a marginally higher protein and carbohydrate intake and showed comparable efficacy to the classical ketogenic diet [16]. It may also be utilized where there is concern about hypertriglyceridaemia

#### Modified ketogenic diet (MKD)

**Standard procedure**: A more relaxed way of giving the ketogenic diet. Carbohydrate intake is restricted to 10 g/day. The intake of fat is actively encouraged. Protein intake is not restricted. This means that during cooking, weighing is restricted to food components containing carbohydrates, while the rest of the food components can be added freely. Carbohydrate-free foods can be offered to the patients unrestricted during the day.
**Contraindications**: none
**Complications**: constipation (46 %), anorexia (18 %), lethargy (6 %), vomiting (10 %) [7••]

Special points: Less restrictive alternative to the traditional ketogenic diet. More suitable for older individuals and may improve compliance in long-time treatment.

#### Low glycemic index treatment (LGIT)

**Standard procedure**: High glycemic index (GI > 50) carbohydrates are eliminated from the diet and total carbohydrates are limited to 40–60 g/day (approx. 10 % of daily calories). Recommended goals for daily protein and fat intake are set to ensure that the patient’s caloric needs are met. Increased fat intake is still encouraged aiming for a 20 % protein, 50–60 % fat diet.
**Contraindications**: none
**Complications**: minimal fatigue, vomiting, and lethargy have been reported [17]

Special points: A less restrictive alternative to the traditional ketogenic diet might improve compliance in long-time treatment. Good efficacy has been reported in children with drug resistant epilepsy (66 % had seizure reduction >50 % over 12 months [17]), but no randomized controlled trials are available.

### Pharmacologic treatment

- Antiepileptic drugs (AED) still are the foundation of epilepsy therapy. This is justified by their efficacy. Of individuals with newly diagnosed epilepsy, 50 % will become seizure-free on a modest or moderate dose of their first antiepileptic drug and another 10 % will be controlled on their second or third drug [1]. Unfortunately, the remaining patients are at risk of developing drug-resistant epilepsy, and their overall percentage has failed to reduce with the introduction of the new (third generation) AEDs [18], despite some gains in tolerability and options for rational polytherapy.
- Since the serendipitous discovery of the anticonvulsant properties of phenobarbital in 1912, the development of new AEDs has continued to be largely based on screening newly synthesized agents for their anticonvulsive effects using animal models for convulsive or nonconvulsive seizures. Thus, we are mainly developing drugs targeting seizures rather than the underlying pathology causing the patient’s seizures. This has been problematic owing to the fact in many the underlying cause of the epilepsy has been unknown.
- Below we will consider the evidence regarding newer pharmacological agents used in childhood epilepsies (stiripentol, zonisamide, perampanel, cannabidiol)

#### Stiripentol

**Background**: Stiripentol was first studied as an add-on AED in a large exploratory observational trial in 1999 showing a surprising favorable response especially in patients with Dravet syndrome. These results were confirmed in two randomized placebo-controlled syndrome-dedicated trials investigating the efficacy of stiripentol as adjunctive therapy to clobazam and sodium valproate [19, 20]. In these two studies, adding stiripentol reduced overall seizure rate by 70 %. There is evidence suggesting that stiripentol acts both through an intrinsic GABAergic effect and through pharmacokinetic interaction by inhibiting cytochrome P450 enzymes [21]. Stiripentol was designated an orphan drug by the European Medicine Agency (EMA) in 2001 and obtained marketing authorization in 2007 “for use in conjunction with clobazam and sodium valproate as adjunctive therapy of refractory generalized tonic-clonic seizures in patients with severe myoclonic epilepsy in infancy (SMEI, Dravet’s syndrome)
**Standard dosage**: initially 10 mg/kg in 2–3 divided doses; titrate dose over minimum of 3 days to max. 50 mg/kg/day in 2–3 divided doses (usually in practice introduced more cautiously).
**Contraindications**: history of psychosis.
**Main drug interactions**: increases plasma concentration of clobazam, carbamazepine, fosphenytoin, phenobarbital, phenytoin, primidone, sodium valproate
**Main side effects**: nausea, vomiting, aggression, anorexia, ataxia, drowsiness, dystonia, hyperexcitability, hyperkinesia, hypotonia, irritability, sleep disorders, weight loss, neutropenia; less commonly fatigue, photosensitivity, rash, and urticaria.

Special points: The combination of stiripentol, clobazam, and sodium valproate is currently considered optimal therapy in patients with Dravet syndrome with refractory generalized tonic-clonic seizures.

#### Zonisamide

**Background**: Zonisamide is a benzisoxazole derivate acting through inhibition of Na + channels and reduction of T-type Ca2+ currents. Following a phase III, double-blind, randomized, placebo-controlled, multicenter trial showing that it is significantly more effective than placebo in controlling partial seizures in children already receiving one or two other AEDs [22•]. It was recently approved for the adjunctive treatment of partial seizures (with or without secondary generalization) in children ≥6 years in Europe. Its safe use in children has been further confirmed in a pooled analysis of 17 studies including 398 pediatric patients ≤16 years treated with zonisamide [23].
**Standard dosage**: initially 1 mg/kg once daily for 7 days, then increased by 1 mg/kg every 7 days; usual maintenance, body weight 20-55 kg, 6–8 mg/kg once daily (max. 500 mg once daily), body weight over 55 kg, 300–500 mg once daily.
**Contraindications**: hypersensitivity to sulfonamides, concomitant use of drugs that increase risk of hyperthermia or metabolic acidosis.
**Main drug interactions**: potent inducers of cytochrome P450 enzyme CYP3A4 such as carbamazepine, phenytoin and phenobarbital increase zonisamide clearance and may necessitate dose increase.
**Main side effects**: decreased appetite, somnolence, fatigue, dizziness, decreased weight, renal stones, irritability and headache. Patients aged 6–11 years (5 % of studied patients): decreased appetite, somnolence, fatigue, irritability and lethargy; while in those aged 12 to16 years, there was decreased appetite, fatigue, somnolence, decreased weight, dizziness, headache, and insomnia [23].

Special points: Increase dose at 2-week intervals in patients who are not receiving concomitant carbamazepine, phenytoin, phenobarbital, or other potent inducers of cytochrome P450 enzyme CYP3A4. Carefully monitor weight of pediatric patients treated with zonisamide.

#### Perampanel

**Background**: One of the principles of rational polytherapy in the epilepsies is the combination of drugs with different, rather than the same, mechanisms of action. Perampanel is the first drug in a novel class of AEDs, as it is a selective noncompetitive AMPA-receptor antagonist [24] and thus is a valuable option as an add-on therapy in a rational polytherapy concept. The AMPA-type glutamate receptors modulate the generation and spread of epileptiform activity by binding glutamate in the post-synaptic excitatory synapses [24]. In a double-blind, placebo-controlled trial of perampanel as an add-on therapy in 143 adolescents (12–17 years) with drug resistant partial seizures perampanel showed sustained seizure frequency improvements and a generally favorable safety profile [25]. Data on efficacy and tolerability in a pediatric population also including younger children was published by Biró et al. The overall responder rate (seizure reduction >50 %) in this retrospective study was 31 % with a better efficacy in children 6 years and older (36 %) than in children under 6 years (9 %) [26].
**Standard dosage**: Child 12–18 years: initially 2 mg once daily before bedtime, increased according to response and tolerability in 2-mg steps at intervals of at least 2 weeks; usual maintenance 4–8 mg once daily; max. 12 mg once daily.
**Contraindications**: severe hepatic impairment, moderate and severe renal impairment.
**Main drug interactions**: Plasma concentration of perampanel reduced by carbamazepine, phenytoin, fosphenytoin, oxcarbazepine and topiramate, and increased by ketoconazole. Perampanel reduces plasma concentration of midazolam, increases plasma concentration of oxcarbazepine and accelerates metabolism of progestogens.
**Main side effects**: nausea, changes in appetite, weight increase, aggression, dizziness, drowsiness, dysarthria, gait disturbance, irritability, anxiety, confusion, suicidal ideation and behaviour, malaise, ataxia, back pain, vertigo, blurred vision, and diplopia.

Special points: Perampanel is the first noncompetitive AMPA-receptor, a novel class of AEDs. It is licensed as an add-on therapy for patients over 12 years with drug-resistant focal seizures.

#### Cannabidiol (CBD)

**Background**: Cannabis has historically been used to treat epilepsy. Recently, the interest for the antiepileptic properties especially of cannabis’ nonpsychoactive compound cannabidiol (CBD) has been rekindled in particular as an add-on therapy for the treatment of the potentially devastating epileptic encephalopathies Dravet syndrome, Lennox-Gastaut syndrome (LGS) and infantile spasms (IS). Pharmacologic studies have proposed TRP channels, G-coupled protein receptor protein 55 (GPR55), or voltage-dependent anion-selective channel protein 1 (VDAC1) as possible targets through which CBD reduces neuronal excitability and neuronal transmission [27]. Preclinical studies have shown CBD to have antiepileptiform and anticonvulsant effects in vitro and in vivo models [27]. There are several publications of parental reports on the efficacy of cannabidiol-enriched cannabis extracts for children with Dravet syndrome, LGS or IS. These report good results with an overall responder rate of 85 % with 14 % reporting complete seizure freedom [28], 84 % with 11 % reporting complete seizure freedom [29] and 33 % (>50 % reduction in seizures) with a much higher responder rate of 89 % for the LGS patients [30]. According to the parent surveys, CBD seemed to be tolerated well by the patients and some reported added beneficial effects on sleep, alertness and mood. These studies, however, are small, and are prone to bias. Randomized clinical trials are required to evaluate efficacy of CBD in children with epilepsy as well as to assess safety and appropriate dosing. Such studies are currently underway for patients with Dravet syndrome and LGS.
**Dosage**: currently ongoing trials in progress to determine optimal dose and tolerability.
**Side effects**: as above.

Special points: CBD seems a promising add on therapy for patients with devastating epileptic encephalopathies, but efficacy and safety have yet to be proven in randomized clinical trials.

### Emerging therapies

- In recent years, next generation sequencing has proven highly effective in revealing gene mutations causing genetic epilepsies [31•, 32]. This has lead to functional studies eliciting the mechanism interrupted by gene mutations and consequently to the identification of potential targets for drug development. This development constitutes a paradigm-changing approach to epilepsy therapy making targeted, rather than empiric treatment a realistic prospect.
- Below, we will discuss the pharmacologic agents currently considered for the treatment of specific genetic epilepsies. None of those treatments are yet validated in the clinical setting.

#### Quinidine in KCNT1-related epilepsies

**Background**: KCNT1 encodes a weak voltage-dependent and intracellular sodium-activated potassium channel. Gain-of-function mutations in the KCNT1 gene have been found to cause two distinct epilepsy phenotypes. Autosomal dominant nocturnal frontal lobe epilepsy (ADNFLE) [33•] and epilepsy of infancy with migrating focal seizures (EIMFS) [34, 35]. Milligan et al. [36•] examined the electrophysiological properties of KCNT1 mutations in Xenopus oocytes and found all mutated channels to yield larger currents than the wild type. Mutations found in patients with ADNFLE were associated with currents that were approximately three times greater than the wild type, while the mutations underlying the more severe phenotype of EIMFS were associated with currents five times greater. The group then examined the effect of the anti-arrhythmic drug quinidine, known to block KCNT1 channels [37], on the mutated channels and found it to normalize potassium conductance. Based on this knowledge Bearden et al. treated a drug resistant patient with EIMFS with quinidine and reported a dramatic reduction in seizure frequency and some developmental improvement [38]. A further two patients with KCNT1 mutations treated with quinidine were recently published by Mikati et al. Again, the patient with EIMFS showed dramatic reduction in seizure frequency, while the other with a novel phenotype showed no improvement [39].
**Dosage**: In the case report mentioned, the patient was started on a dose of 2 mg/kg/day followed by a 4 day dose titration up to 33 mg/kg/day divided into four doses. After 6 weeks, the dose was further increased to 42 mg/kg/day due to seizure recurrence. Serum quinidine levels ranged from 1.5–4 μg/ml (typical therapeutic range for arrhythmias = 2–5 μg/ml and toxicity is thought to occur at levels >6 μg/ml).

Special points: quinidine is not licensed as an AED.

#### Retigabine in KCNQ2-related epilepsies

**Background**: KCNQ2-related disorders represent a continuum of overlapping neonatal epileptic phenotypes caused by a heterozygous mutation in KCNQ2, the gene encoding the potassium voltage-gated channel subfamily KQT member 2, also known as Kv7.2. The clinical features of KCNQ2-related disorders range from KCNQ2-related benign familial neonatal epilepsy (KCNQ2-BFNE) at the mild end to KCNQ2-related epileptic encephalopathy (KCNQ2-NEE) at the severe end [40]. Retigabine is an AED currently used as adjunctive therapy in adults with partial-onset seizures and is known to selectively enhance the function of potassium channels formed by neuronal Kv7 subunits [41]. Experimental studies have shown retigabine [42] as well as retigabine derivative HN38 [43] to significantly increase potassium currents in mutated KCNQ2 channels, suggesting their potential as a targeted therapy for KCNQ2-related epilepsies. Interestingly the KCNQ2-related epilepsies have recently been shown to respond particularly well to drugs known to act on sodium channels including carbamazepine and phenytoin, leading to them being proposed as first line treatment in these epilepsies [44].

#### Fenfluramine in Dravet syndrome

**Background**: Dravet syndrome, a severe childhood epilepsy characterized by persistent, often drug-resistant seizures and progressive developmental delay is known to be caused by mutations in the SCN1A gene encoding the voltage-gated sodium channel subunit alpha NA_V_1.1. Fenfluramine is an amphetamine like drug that was launched for obesity in the 1990s. Due to cardiac side effects (eg pulmonary hypertension and heart valve disease) it was withdrawn from the market in 1997. The drug acts through inhibition of serotonin uptake and by release of serotonin due to a disruption of vesicular storage. Because of the high density of serotonin receptors in structures critically involved in epilepsies, such as the hippocampus, fenfluramine is believed to have an antiepileptic effect. In a recent study by Zhang et al., it was proven to reduce epileptiform activity in zebrafish larvae with an antisense knockdown of scn1Lab, which is the zebrafish analog to SCN1A [45]. After an earlier report by Boel et al. in 1996 [46], Ceuleman et al. trialed fenfluramine as an add-on treatment in 12 patients with Dravet syndrome over 1–19 years with remarkable results. Of the 12 patients, 8 were seizure-free at their last follow up visit. Overall patients were seizure-free for a mean of 6 (1–19) years. No serious adverse events occurred [47••]. Clinical trials are planned to evaluate the effect of fenfluramine in a randomized controlled setting.

#### Memantine in GRIN2A-related epilepsies

**Background**: Mutation in the GRIN2A gen encoding the GluN2A subunit of the NMDA receptorss have been associated with several childhood-onset epilepsy syndromes including disorders of the epilepsy-aphasia spectrum as well as some described cases of early onset epileptic encephalopathy. The NMDAR antagonist memantine was found to in vitro inhibit mutant channels with the mutation c.2434C > A, resulting in a leucine to methionine substitution at residue 812. Administration of the drug to a patient carrying this exact mutation led to a decrease in seizure frequency and allowed for tapering of conventional AEDs. A different GluN2A mutation (N615K) was proven sensitive to another NMDAR antagonist indicating a need for specific evaluation of each mutant GRIN2A to evaluate response to NMDAR antagonists [48]. Memantine is yet to be trialed clinically.
